# Case report: Successful outcome of treatment using rituximab in an adult patient with refractory minimal change disease and β-thalassemia complicating autoimmune hemolytic anemia

**DOI:** 10.3389/fmed.2022.1059740

**Published:** 2022-11-03

**Authors:** Jing Zhuang, Zhigang Zhao, Changrong Zhang, Xue Song, Chen Lu, Xuefei Tian, Hong Jiang

**Affiliations:** ^1^Division of Nephrology, Department of Internal, People’s Hospital of Xinjiang Uygur Autonomous Region, Urumqi, China; ^2^Division of Nephrology, Department of Internal Medicine, The First Affiliated Hospital of Xinjiang Medical University, Urumqi, China; ^3^Section of Nephrology, Department of Internal Medicine, Yale University School of Medicine, New Haven, CT, United States

**Keywords:** refractory nephrotic syndrome, minimal change disease, β-thalassemia, rituximab, autoimmune hemolytic anemia

## Abstract

Minimal change disease (MCD) is one of the common causes of idiopathic nephrotic syndrome (INS), accounting for 10–20% of INS in adults. Glucocorticoids are the most commonly used and effective drugs in the treatment of MCD, but there is still a proportion of adult patients with MCD who are characterized by glucocorticoid resistance, glucocorticoid dependence, and frequent relapse, which are defined as refractory nephrotic syndrome. Glucocorticoid combination with immunosuppressants is frequently used in patients with refractory nephrotic syndrome, and patients concerned about adverse effects caused by long-term high-dose glucocorticoid therapy. Recent studies have suggested that Rituximab (RTX), a chimeric monoclonal antibody targeted against the pan-B-cell marker CD20, combined with a small or medium dose of glucocorticoid has a beneficial effect with less adverse effects on adult patients with refractory MCD. β-thalassemia is an inherited hemoglobulin disorder caused by the mutation of genes that encode β-globin and results in ineffective erythropoiesis. We here report a case of an adult patient with refractory MCD complicated with β-thalassemia minor accompanied by autoimmune hemolytic anemia (AIHA). MCD relapsed several times despite treatment using glucocorticoid combined with or without different immunosuppressive agent regimens. The β-thalassemia minor was caused by heterozygosity for a 4-base deletion mutation [codons 41/42 (−TTCT) BETA^0^] of the β-globin gene. After the administration of RTX, MCD achieved clinical complete remission, and the anemia due to mild β-thalassemia recovered to normal as well. The disease situation remained stable during 36 months of follow-up. These findings suggest that RTX may contribute to the improvement of refractory MCD and anemia in β-thalassemia minor accompanied by AIHA.

## Introduction

Minimal change disease (MCD) is one of the common causes of idiopathic nephrotic syndrome (INS) in adults, with the use of glucocorticoids being the mainstay. Although the use of glucocorticoids is generally effective in the treatment of adult patients with MCD, there are many challenges in clinical practice such as frequent relapse of kidney disease, glucocorticoid resistance, severe adverse effects induced by glucocorticoids, etc. (1). MCD is a glomerular disease characterized by podocyte injury. The pathological features of the kidney generally show normal glomerular structure under the light microscope and diffuse effacement of the podocyte foot process under the scanning electron microscope (2, 3). The response rate of adult patients with MCD to glucocorticoid treatment has been reported approximately 75% (4). While long-term glucocorticoid treatment can cause adverse effects even life-threatening outcomes. With Rituximab (RTX), a chimeric monoclonal antibody targeted against the pan-B-cell marker CD20, successful application in many immune-mediated proteinuric glomerular diseases such as primary membranous nephropathy (3), patients with glucocorticoid-dependent or glucocorticoid-resistant MCD have been tried to treat using RTX and promising improvement in these patients have been observed (3, 5). The mechanism of action of RTX on the MCD may be mediated by the depletion of CD20^+^ B lymphocytes and reconstruction of abnormal Th17/Treg cell balance (6).

β-thalassemia is one of the most common inherited HGB diseases in the red blood cells, that is caused by abnormal β-globin genes (*HBB*) and HGB synthesis (7). Depending on the affected numbers of *HBB* and the severity of the anemia, β-thalassemia has been classified into three types consisting of the minor (trait), intermedia, and major types. Patients with heterozygous β thalassemia, namely minor type, usually present no symptoms of anemia except complicating the autoimmune hemolytic anemia (AIHA) (8). Here, we report an adult female patient with refractory MCD and β-thalassemia complicating AIHA who has been treated with RTX, both of the symptoms of kidney disease and anemia have been greatly improved and reached complete remission, and remained stable during 36 months of follow-up. These findings suggest that the RTX treatment may be a potential therapeutic strategy for adult refractory MCD and/or AIHA.

## Case presentation

**FIGURE 1:**
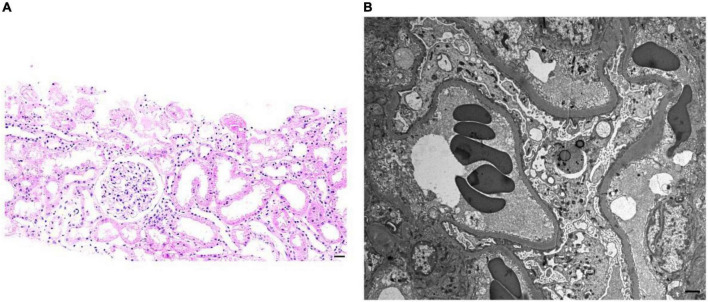
Representative images of light microscopy and transmission electron microscopy (temtem) from the kidney biopsy of the patient. **(A)** Hematoxylin and eosin staining showed no significant changes in glomeruli, renal interstitium, and renal tubules (scale bar: 100 μm). **(B)** Representative TEM showed the diffuse foot process effacement of podocytes, and no electron-dense deposition in the subepithelial, subendothelial, and mesangial compartments was detected (scale bar: 1 μm).

A 22-year-old Chinese woman came to the hospital due to persistent fatigue in 2016. The complete blood count (CBC) results showed normocytic normochromic anemia and hemoglobin (HGB) levels were 89 g/L. She was diagnosed with β-thalassemia without specific treatment for anemia at that time. In the same year, she was referred to our hospital due to sudden peripheral edema in both lower legs and a recent 2-kg increase in body weight, proteinuria, and hypoalbuminemia, and she was diagnosed with nephrotic syndrome. Her past medical history was not significant. She had regular menstruation and normal eating habits. The findings of the physical examination showed normal blood pressure with 120/70 mmHg, mild anemia, mild pitting edema of both lower limbs, and no rash or arthralgia. Laboratory tests results showed that 2 + of urine protein, hypoalbuminemia (28 g/L), normal range of serum creatinine levels; normocytic normochromic mild anemia, positive Coombs test, reticulocytosis, without any positive signs on the liver, spleen and systemic superficial lymph nodes. The level of serum lactate dehydrogenase (LDH) was normal (233 U/L, normal range in our hospital: 109–245 U/L). The patient was further examined excluding common secondary causes leading to anemia such as systemic lupus erythematosus (SLE), anti-neutrophil cytoplasmic antibody-associated vasculitis (AAV), hepatitis B virus infection, hepatitis C virus infection, and human immunodeficiency virus (HIV) infection, paroxysmal nocturnal hemoglobinuria, etc. A renal biopsy was performed to pathologically analyze the cause of the nephrotic syndrome which showed the MCD (Figure 1).

**FIGURE 2:**
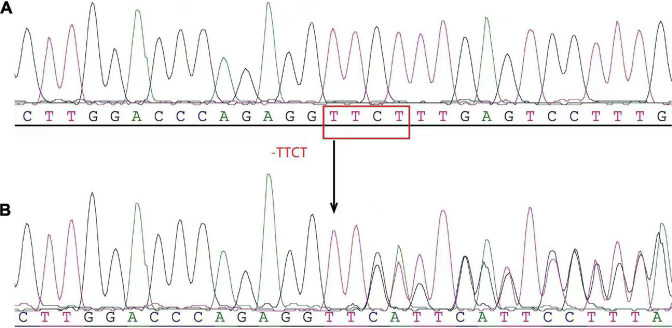
The heterozygosity for the 4-base deletion mutation [codons 41/42 (–ttct) bETa^0^] of the β-globin gene (*hbb*) was determined by high-throughput gene sequencing. **(A)** Normal control reference sequence. **(B)** Patient. The arrows indicated the site of the deletion mutation of the β-globin gene.

To further investigate the underlying cause of anemia, after discussion with the patient and obtaining her consent, the peripheral mononuclear cells were isolated from the blood for a gene test described as in our previous study (9). In brief, Genomic DNA was extracted from peripheral blood samples using the blood genomic DNA Extraction Kit and the Thermo Scientific KingFisher Flex magnetic Bead Purification System. The α-globin genes (*HBA*) and *HBB* were detected by high-throughput sequencing. Four deletion types of alpha-thalassemia (−−^SEA^, −α^3^.^7^, −α^4^.^2^, and −−^THAI^) and three deletion types of β-thalassemia [Chinese Ggamma (Agammadeltabeta)0, SEA-HPFH, Taiwan type] were detected by gap-PCR. Meanwhile, more than 153 mutation types of α-thalassemia and more than 348 mutation types of β-thalassemia were detected as well. PCR amplification was performed using TaKaRa PCR amplification reagent 2 × GC Buffer I and TaKaRa Taq™ Hot Start Version (BGI Diagnosis Co., Ltd., Shenzhen, China). Analysis results revealed heterozygosity for Codons 41/42 (−TTCT) BETA^0^ of the β-globin gene (Figure 2). The patient had no siblings. Her parents presented normal HGB levels and declined the request for the gene test.

**FIGURE 3:**
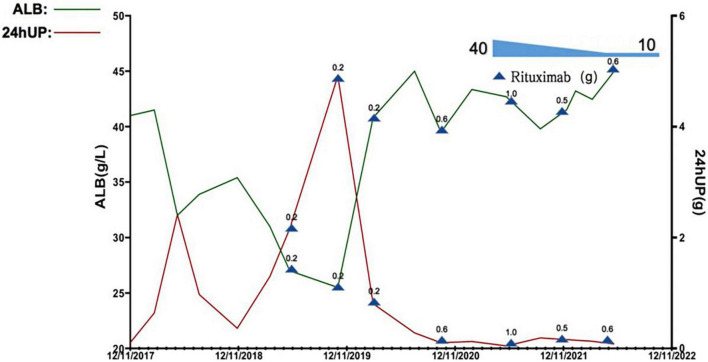
Quantification in levels of plasma albumin (alb) and 24-h urinary protein excretion (24hUP) of this patient during the observational period. 0.2, 0.5, 0.6, and 1.0 denote the dose of rituximab used at this timepoint (g, gram).

According to the renal histopathological changes, treatment with oral prednisolone acetate at a dose of 40 mg/day was initiated, and clinical complete remission for the nephrotic syndrome was achieved after 4 weeks of treatment based on the criteria recommended by The Kidney Disease: Improving Global Outcomes (KDIGO) Clinical Practice Guideline for the Management of Glomerular Diseases in 2021 (10). However, kidney disease recurred during the gradual taper of the glucocorticoid dose. Given that, the treatment regimen was changed to prednisolone acetate at a dose of 20 mg/day in combination with mycophenolate mofetil (MMF) at a dose of 1.5 g/day by mouth to control the kidney disease, during which the levels of 24-h urinary protein excretion decreased. In March 2017, kidney disease recurred without the defined causes. The therapeutic regimen was adjusted to prednisolone acetate at a dose of 10 mg/day in combination with the calcineurin inhibitor tacrolimus (FK506) at a dose of 2 mg/day, the trough concentration of tacrolimus was maintained at 4.2 ng/ml. After 1 month of treatment, partial clinical remission of nephrotic syndrome was achieved. However, kidney disease recurred again after 1 year without any defined cause, and the treatment regimen was changed to prednisolone acetate combined with oral cyclophosphamide by mouth. The MCD relapsed 5 times totally during the dose taper of prednisolone acetate. After discussion with the patient, the therapeutic regimen was adjusted to multi-target treatment using prednisolone acetate at a dose of 25 mg/day, MMF, and FK506. Partial remission of the nephrotic syndrome was reached with a trough level of FK506 of 2.7–6.9 ng/ml and a cumulative dose of 5.8 g for cyclophosphamide (Figure 3). Of note, the HGB levels for this patient kept low levels without any change during the treatment. There was no gastrointestinal bleeding or increased menstrual volume. The results of ferritin and transferrin saturation excluded iron deficiency anemia. She has no allotriophagia habit and no past medical history of blood transfusion. The results of kidney function showed normal. The anemia caused by chronic loss of red blood cells, impairment of kidney function, and side effects of drugs were excluded. The result of the CBC showed no significant abnormalities in white blood cell count and platelet count. The Coombs test was still positive. The request for a bone marrow aspiration or biopsy was declined by the patient. These findings revealed that the mild anemia may be caused by β-thalassemia minor combined with autoimmune hemolysis.

The adverse effects associated with long-time use of glucocorticoids including skin acne, and osteoporosis emerged in this patient. Considering the patient’s refractory MCD and β-thalassemia minor complicating with AIHA, detailed discussions regarding the diseases and the adverse effects of long-term use of glucocorticoid and immunosuppressive agents were conducted with the patient. With the permission of the patient, the CD20^+^ B-cell depletion biological agent RTX combined with prednisolone acetate at the dose of 25 mg/day plus FK506 at a dose of 0.5 mg twice a day was administered starting from September 2019.

**FIGURE 4:**
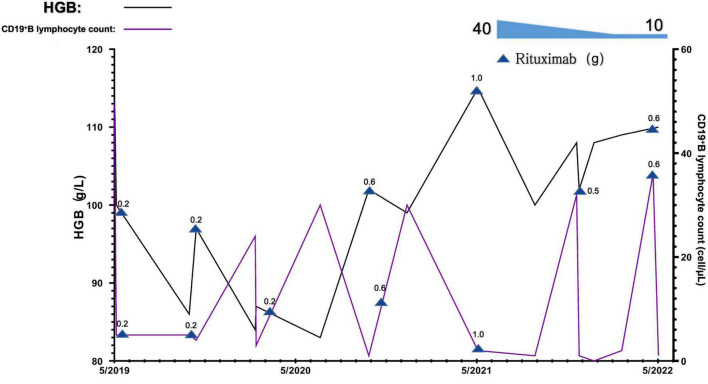
Changes in blood hemoglobin (hgb) levels and CD19^+^B lymphocyte counts of this patient during the observational period. 0.2, 0.5, 0.6, and 1.0 denote the dose of rituximab used at this timepoint g, gram.

The RTX dose and schedule used in this patient were guided by the serum CD19^+^ B lymphocyte counts, immunological status, evaluation of potential infection risk, and the response to the RTX treatment. The cut-off of serum CD19^+^ B lymphocyte counts was 5/μl. Eight doses of RTX were given as follows, 200 mg on September 2019, 500 mg on November 2019, 200 mg on March 2020, 500 mg on July 2020, 600 mg on January 2021, 1,000 mg on June 2021, 500 mg on Dec 2021, and 600 mg on June 2022. The glucocorticoid has been tapered and withdrawn in the end. The serum CD19^+^ B lymphocyte counts were significantly decreased. The parameters associated with refractory MCD including increased 24-h urine protein excretion and hypoalbuminemia, and anemia significantly improved 6 months after treatment, the Coombs test turned negative. The serum CD19^+^ B lymphocyte counts gradually reconstructed and returned to normal after 4∼5 months of RTX withdrawal. During the 36-month follow-up observational period, clinical complete remission of refractory MCD and normal blood HGB levels remained stable (Figures 3, 4).

## Discussion

Here we reported a case of a young Chinese woman with refractory MCD and β-thalassemia minor complicating AIH. According to the 2021 KDIGO guideline for glomerular disease, adequate glucocorticoids are recommended for the adult patient with MCD treatment, combined immunosuppressive agents are recommended if necessary (10). The patient was treated with a series of recommended therapeutical regimens for refractory MCD using glucocorticoids combined with other immunosuppressive agents. The clinical outcome was not satisfactory, whereas the adverse effects of the drugs emerged. Long-term use of glucocorticoids in the patient can cause unavoidable adverse effects, mainly manifested as infection, skin acne, osteoporosis, increased psychological burden, and decreased quality of life (9). In order to reduce the adverse effects caused by glucocorticoids and other immunosuppressive agents, and favor the recovery of refractory MCD, the RTX was administered to this patient. Notably, both kidney disease and anemia caused by β-thalassemia minor complicating AIH reached complete remission and remained stable during the 36-month follow-up observational period.

The pathogenesis of MCD remains elusive although the understanding of the underlying pathogenic mechanisms for MCD has recently made a tremendous leap. Studies indicate that the pathogenesis of MCD is closely related to immune disorders, and may be affected by genetic factors and environmental factors (3, 11). Activation of T cells plays an important role in the occurrence and development of MCD. Burgeoning studies have shown that the involvement of B cells in activating T cells may also play a role in the pathogenesis of MCD. In addition to the synthesis of specific antibodies stimulated by antigens, B cells are also involved in antigen presentation and costimulatory signals and secrete cytokines to regulate the differentiation of T cells (12). Recently, the use of RTX in children and adults with refractory nephrotic syndrome has achieved promising efficacy, which further suggests that B cells may play an important role in the pathogenesis of MCD (5). RTX is a chimeric monoclonal antibody targeted against the pan-B-cell marker CD20 molecule with mouse variable and human constant region. By binding to CD20 molecules on the surface of B cells, RTX causes rapid depletion of CD20^+^ B cells through complement-dependent cytotoxicity, antibody-dependent cell-mediated cytotoxicity, and induction of apoptosis (13). Because B cells can activate the antigen presentation function of T helper cells (14), the depletion of B cells regulates the immunological function of T cells and reduces the secretion of various cytokines and circulating factors. The underlying mechanism of RTX on the inhibition of B cell growth or promotion of B cell apoptosis may be mediated by its activation of protein kinase and phospholipase Cγ (15). Additionally, RTX can enhance CTLA4 (Cytotoxic T-lymphocyte Associated Protein 4) production by regulatory T cells (Treg), thereby inhibiting CD80 activation and signaling pathways between antigen-presenting cells and T cells, which favors the reconstruction of immune disorders in glomerulus and reduction of proteinuria (11). Recent small-size sample studies have shown that RTX can rapidly mitigate the nephrotic syndrome in adult MCD and maintain a longer remission (11, 16). These findings suggest that RTX may be a safe and effective alternative to glucocorticoids and other immunosuppressive agents in patients with a long history of relapsing refractory MCD. The adult female patient with refractory MCD reported here responded very well to eight times RTX treatment as needed and no adverse events were observed during the whole 36-month follow-up observational period. Similar studies using more than two doses of RTX for the successful treatment of adult patients with frequently relapsing MCD have been recently reported (5, 17). The mechanism of the RTX treatment favoring the remission of MCD remains quite unclear. A 2022 study reported that the serum antibodies against nephrin, an important podocyte-specific protein, have been discovered in some children and adult patients with MCD, the anti-nephrin antibodies presented positive during the active phase and negative during the remission phase after treatment. Meanwhile, confocal microscopy showed the co-localization of immunoglobulin G (IgG) and nephrin in the glomerular podocytes (18). These findings suggest that B cell-associated humoral immunity is also possibly involved in the pathogenesis of MCD. The glomerular podocyte injury is the predominant pathological feature of MCD, which cause the dysfunction of the integrity of the glomerular filtration barrier (3). Nephrin is a 180 KD transmembrane junction protein specifically located on slit diaphragm membranes between adjacent podocyte foot processes, which is a key protein involved in maintaining the podocyte cytoskeletal structure and integrity of the glomerular filtration barrier (19). The recovery of impaired nephrin, either loss or translocation, to normal may serve as a specific marker predicting clinical remission of MCD (20). Recent reports showed that RTX could specifically increase the expression of SMPDL3b (Sphingomyelin phosphodiesterase acid-like 3B) in podocytes, thereby stabilizing the cytoskeleton of podocytes, reducing podocyte apoptosis, and protecting podocyte function (21). Recent studies have shown that RTX can substantially increase the remission rate of MCD in adults with refractory nephrotic syndrome to 70.4% and higher, shorten the time reaching remission to 1–2 months, significantly reduce the dose of glucocorticoid use, and greatly alleviate the recurrence rate of MCD (22), Nevertheless, more convincing evidence and data are required to understand the underlying immune pathogenesis of MCD and efficacy of RTX on MCD.

The patient reported in this study also presented mild anemia caused by β-thalassemia minor complicating with AIHA. The genetic testing result revealed compound heterozygosity for a 4-base deletion mutation [Codons 41/42 (−TTCT) beta^0^] in the HGB β gene *HBB*. The prevalence of thalassemia was 0.46 per 1,000 cases (23). In a recent study of prenatal diagnosis of α-thalassemia and β-thalassemia in 3,049 families in China, the most common mutation for β-thalassemia was the mutation of Codons 41/42, which accounted for 30.27% (24). A previous study reported the rate of patients with β-thalassemia co-existence of autoantibodies or alloantibodies was 38.9% (25). In some patients with β-thalassemia, anti-erythrocyte autoantibodies are present, making the direct antiglobulin test (DAT) positive. In the past, there is no specific treatment regimen recommended for such mild anemia with β-thalassemia patients with autoimmune hemolysis, but the persistent anemia leads to potential harm to the functions of organs and a significant decline in the quality of life of patients. The confirmative diagnosis of β-thalassemia depends on gene tests and DNA analysis. Genetic analysis showed that the patient had compound heterozygosity for the Codons 41/42 (−TTCT) Beta^0^ mutation, which not only resulted in structural abnormality of HGB but also caused mild anemia. To our knowledge by reviewing the published papers available, it is the first time to report the efficacy of RTX treatment on an adult patient with refractory MCD, who also has the β-thalassemia complicated with AIHA caused by Codons 41/42 (−TTCT) BETA^0^ in the *HBB*. However, there is no evidence to support whether the variant of the Codons 41/42 (−TTCT) BETA^0^ in *HBB* of this patient is an inherited or sporadic variation as she has no siblings and her parents refused genetic testing. The favorable effect of RTX on the improvement of anemia in this patient is speculated to be related to β-thalassemia itself, β-thalassemia may directly lead to sustained immune stimulation (26, 27). The research focused on AIHA in patients with β-thalassemia is limited. β-thalassemia is caused by the absent (BETA^0^) or insufficient (BETA^+^) production of the β chain of the HGB. The imbalance of HGB chain synthesis in erythrocytes results in an excess of released α-globulin chains, which precipitate in precursors of erythrocytes and lead to structural changes in the cell membrane (28). The presence of these abnormal erythrocytes results in the continued activation of monocytes responsible for immune clearance, alterations of T and B lymphocytes, etc., that possibly be involved in the pathogenesis of AIHA (27, 29). Alloimmunization in patients with β-thalassemia major is usually associated with multiple blood transfusion (27, 28). AIHA in the patient reported here was not transfusion dependent. The pathogenesis of MCD is also involved in the disorder of the immune system. The underlying mechanisms linking the AIHA and β-thalassemia minor in the current case requires further investigation. A study reported that seven patients with β-thalassemia major and AIHA received corticosteroid treatment and blood transfusion, in whom six patients responded well to the management, and the improved HGB remained stable for more than 6 months of following-up (30). The adult female β-thalassemia minor complicated with AIHA reported in this study did not respond well to the glucocorticoids and other immunosuppressant agents while treating the MCD (Figure 4). However, the use of RTX achieved pronounced efficacy on anemia and greatly improved the quality of life of the patient. The specific mechanism of action needs to be further studied.

In this study, an adult patient with refractory MCD with β-thalassemia minor complicated AIHA was treated with RTX and reached complete remission in both nephrotic syndrome and anemia. It could maintain the patient in a relatively stable remission state after the gradual withdrawal of non-specific immunosuppressants and glucocorticoids. There were no adverse effects reported or observed regarding the use of RTX during the observational periods. However, there are some limitations to this study. There is only one patient reported, and more similar cases need to be collected to verify the efficacy of RTX in such patients. Secondly, the follow-up after management with RTX is not too long, more observational period is required. Meantime, The refractory MCD and anemia caused by the β-thalassemia minor complicated AIHA reached complete remission after using RTX, while the anemia did not respond to the previous treatment with glucocorticoids and immunosuppressants management, what is the potential mechanism of RTX on them? The role of CD20^+^ B lymphocytes in their pathogenesis needs to be further investigated.

In conclusion, the prevalence of refractory MCD with β-thalassemia minor complicated with autoimmune hemolysis seems to be lower than in the general population. We report a case of relatively successful treatment of this type of disease using CD20^+^ B lymphocyte depletion biologic agent RTX, which may potentially provide a new choice for future clinical treatment strategy and mechanism exploration.

## Data availability statement

The datasets presented in this study can be found in online repositories. The names of the repository/repositories and accession number(s) can be found below: The National Omics Data Encyclopedia (NODE), accession number: OEP003683.

## Ethics statement

The studies involving human participants were reviewed and approved by the Medical Ethics Committee of the People’s Hospital of Xinjiang Uygur Autonomous Region. Written informed consent to participate in this study was provided by the participant.

## Author contributions

JZ, HJ, ZZ, CZ, XS, and CL made clinical data collection and actively involved in the clinical care of the patient. HJ and XT made substantial contributions to the research idea and study design. HJ and CZ evaluated the renal pathology of the patients. All authors contributed to important intellectual content during manuscript drafting and revision and accepted accountability for the overall work by ensuring that questions about the accuracy or integrity of any portion of the work are appropriately investigated and resolved and contributed to the article and approved the submitted version.
